# Structure-Guided Immunoinformatics for the Rational Design of a Multi-Epitope Vaccine Against Batai Orthobunyavirus

**DOI:** 10.3390/ph19081307

**Published:** 2026-08-18

**Authors:** Maha Abdullah Alwaili, Nawal Al-Hoshani, Huda A. Alqahtani, Rasha Alonaizan, Khaled Alzhrani, Tariq Aziz

**Affiliations:** 1 Department of Biology, College of Science, Princess Nourah bint Abdulrahman University, P.O. Box 84428, Riyadh 11671, Saudi Arabia; maalwaele@pnu.edu.sa (M.A.A.);; 2 Department of Zoology, College of Science, King Saud University, Riyadh 11495, Saudi Arabia; 3 Faculty of Science, King Saud University, P.O. Box 2455, Riyadh 11451, Saudi Arabia; 4 Department of Clinical Laboratory Sciences, College of Applied Medical Sciences, Jouf University, Sakaka 72388, Saudi Arabia; 5 Biodiversity Genomics Unit, University of Tabuk, Tabuk 71491, Saudi Arabia

**Keywords:** Batai orthobunyavirus, multi-epitope vaccine, envelope glycoprotein, structural modeling, molecular docking, TLR4, biophysical characterization

## Abstract

**Background/Objectives:** Batai orthobunyavirus (BATV) is an emerging mosquito-borne zoonotic pathogen for which no licensed vaccine is currently available. The viral envelope glycoprotein plays an important role in viral attachment and host immune recognition, making it a potential target for rational vaccine design. **Methods:** In this study, an immunoinformatics-based framework was used to design and evaluate a multi-epitope vaccine candidate targeting the BATV envelope glycoprotein. Selected B-cell, cytotoxic T-lymphocyte (CTL), and helper T-lymphocyte (HTL) epitopes were assembled using appropriate linkers and a human β-defensin adjuvant. Population coverage and in silico immune simulations were conducted to evaluate the potential breadth and magnitude of immune response. **Results:** The final vaccine construct demonstrated favorable physicochemical characteristics, high predicted antigenicity (0.7959), and non-allergenic properties while maintaining favorable predicted structural characteristics and broad predicted population coverage (99.92%). Structural docking revealed a stable interaction between the vaccine construct and human TLR4, with a weighted docking score of −1194.8, suggesting favorable molecular recognition and receptor engagement. Normal Mode Analysis further supported the structural stability and conformational integrity of the vaccine receptor complex. Immune simulation predicted robust primary and secondary immune responses characterized by elevated IgM and IgG antibody production, sustained memory cell formation, and strong IFN-γ and IL-2 responses, indicating the potential to elicit balanced humoral and cellular immunity. **Conclusions:** This study presents a structurally optimized and validated multiepitope vaccine candidate against the emerging Batai orthobunyavirus. These computational findings identified a promising vaccine candidate for further investigation; however, its immunogenicity, safety, and protective efficacy before further vaccine development can be considered.

## 1. Introduction

As the number of zoonotic spillovers increases and climate change continues to alter the ranges of viruses, placing them in new areas of the world, BATV has emerged as an increasingly relevant mosquito-borne zoonotic pathogen with expanding geographic distribution and potential public-health significance [1]. However, there is no licensed vaccine or specific antiviral drug to control its effects. Developing a universal preventive measure is also complicated by the genetic diversity of orthobunyaviruses, requiring the selection of antigens with an exceptionally high degree of precision. The present study addresses this gap by applying an immunoinformatics framework to identify candidate epitopes and design a multi-epitope vaccine construct [2,3].

Batai orthobunyavirus, or BATV, is a negative-sense, tripartite RNA virus belonging to the Orthobunyavirus genus of the Peribunyaviridae family. Its primary vectors are the Culex and Anopheles mosquito species, and its distribution includes much of Europe and Asia. Previous studies have linked BATV to “Batai fever” in humans and to significant genetic reassortment events, including the emergence of the highly pathogenic and significant veterinary disease agent, the Schmallenberg virus [4,5]. While the virus causes a benign, self-limiting fever in humans, it is increasingly being implicated as a significant agent of severe reproductive disorders and congenital anomalies in livestock. Despite its increasing range and its associated human health risk, there remain no licensed vaccines or effective antiviral therapies, and mosquito control remains the principal method of prevention worldwide [3,6,7].

In spite of the recognized risks and the expanding footprint of Batai orthobunyavirus, traditional approaches to vaccine development are limited by the high costs, time requirements, and safety concerns associated with live-attenuated or killed virus production [8]. Previous efforts have focused on surveillance and phylogenetic tracking, with a limited availability of targeted preventive strategies existing for the design of targeted, safe, and effective preventive measures. Complicating matters further are the genetic complexities of orthobunyaviruses, which suggest that a single-antigen vaccine will not provide effective cross-protection for diverse human populations. The current study seeks to address this void by taking a computational immunoinformatics approach to design the BATV proteome, going beyond traditional approaches to design a chimeric multi-epitope vaccine for safety and global population coverage [9].

The present study used an immunoinformatics framework to detect immunogenic epitopes and design a multi-epitope vaccine candidate for BATV. The antigenicity, physicochemical properties, structures, receptor interaction, population coverage, and simulated immune response to the construct were computed. The selected epitopes predicted to have a global population coverage of 99.92%, suggesting theoretical HLA representation. However, all the results presented in this work are computational results and need experimental confirmation to demonstrate the immunogenicity, safety, and efficacy of the proposed vaccine candidate.

## 2. Results

### 2.1. Allergenicity and Antigenicity Prediction

The antigenicity of the glycoprotein was predicted using VaxiJen v2.0 (virus model, threshold 0.4), which revealed a score of 0.5004, classifying the glycoprotein as a “Probable Antigen” and supporting its selection for subsequent epitope mapping. Safety prediction using the AllerTOP v2.1 webserver revealed the glycoprotein as a “Probable Non-Allergen,” implying a low likelihood of inducing a type I hypersensitivity response in the host. Therefore, the glycoprotein emerges as a strong candidate for the induction of a strong immune response and its subsequent use as a vaccine candidate for B-cell and T-cell epitope mapping.

### 2.2. Topology, Signal Peptide, and Physicochemical Characterization

**Figure 1:**
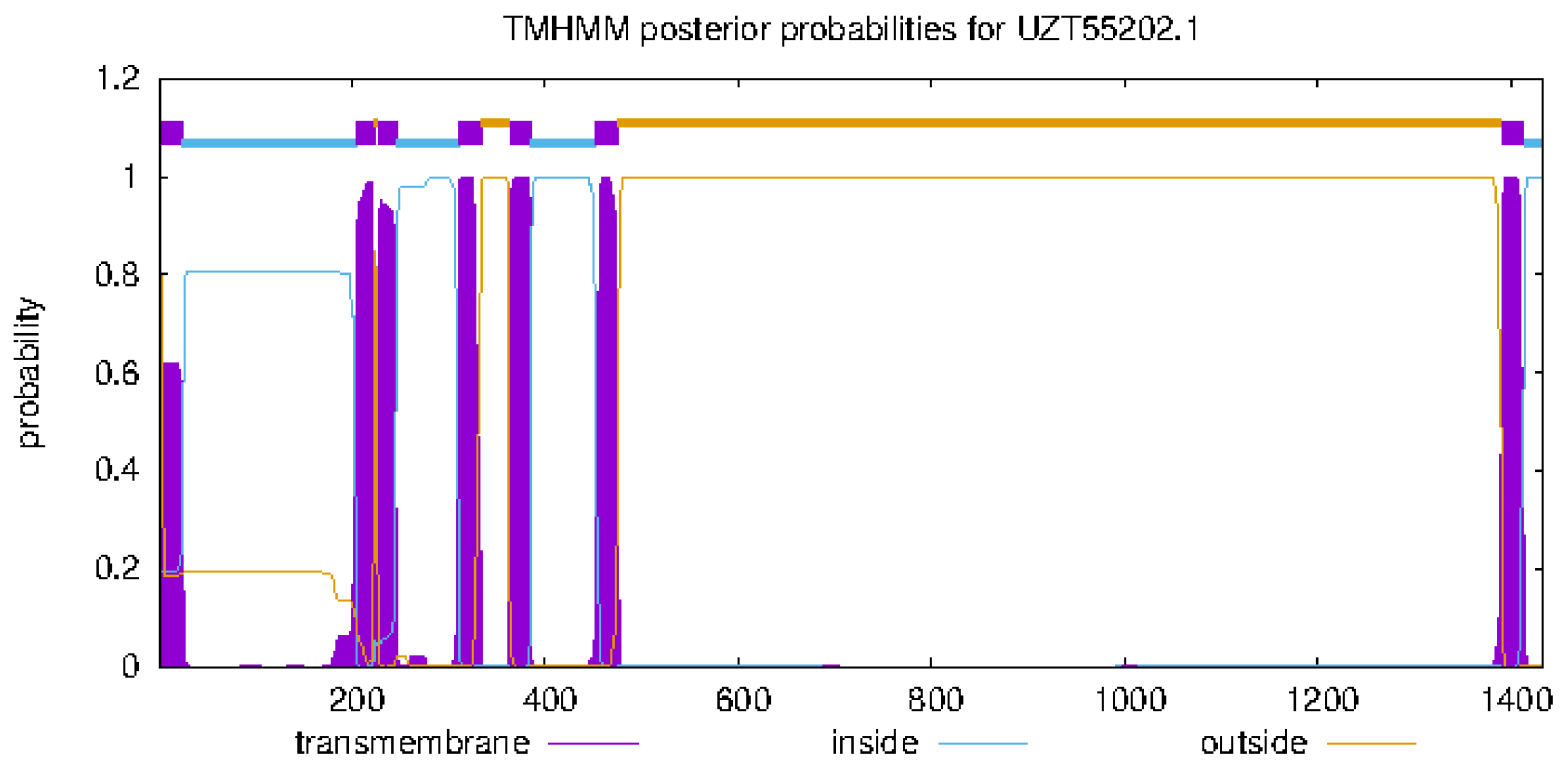
The presence of transmembrane helices in the candidate glycoprotein for vaccine design.

Using the TMHMM V2.0 prediction server, the orientation of the BATV glycoprotein (UZT55202.1) in the membrane was determined. The prediction indicated that the glycoprotein contains seven transmembrane helices. The helices are located in the regions of amino acid positions 4–23, 205–222, 227–246, 311–333, 363–385, 453–475, and 1391–1413. The glycoprotein has a large external region outside the cell membrane, extending from positions 476–1390, making it highly favorable as a vaccine target, as it is easily accessible by B-cells and T-cells. Figure 1 below represents the presence of transmembrane helices in the candidate protein for vaccine design.

**Figure 2:**
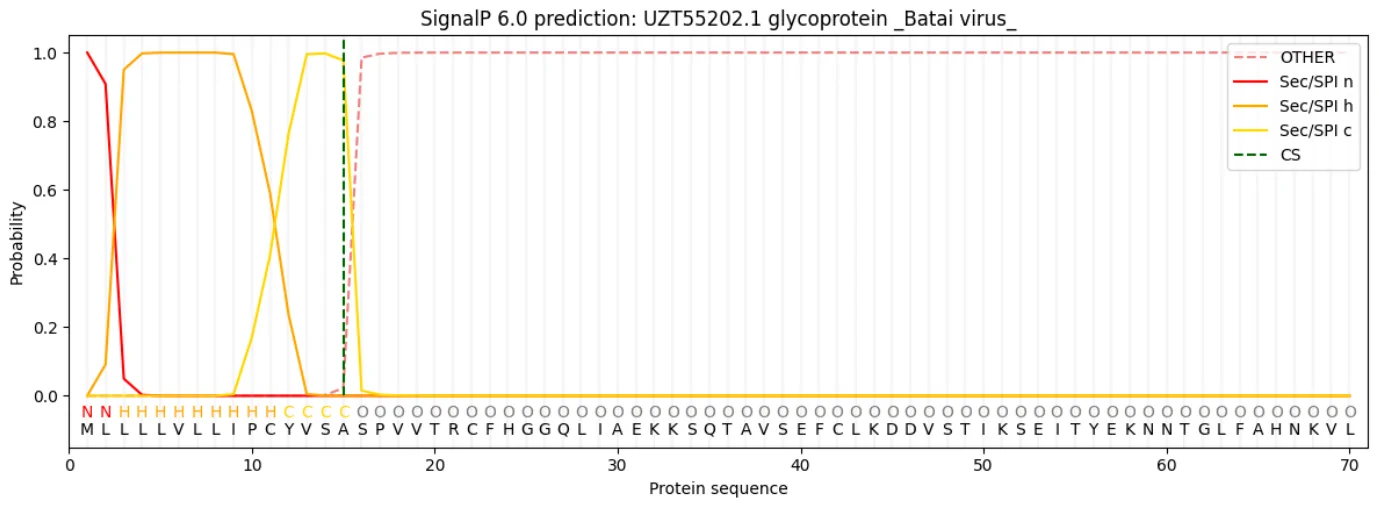
The presence of signal peptide regions in the candidate glycoprotein for vaccine design.

To determine the secretory potential of the glycoprotein, the sequence was submitted for prediction using the SignalP 6.0 prediction server. The prediction showed that the glycoprotein has a very high signal peptide (Sec/SPI) with a high score of 0.9991. The cleavage site is located between amino acid positions 15 and 16 with a probability of 0.9774. The signal peptide is consistent with the glycoprotein’s secretory pathway as a surface-attached viral attachment protein. Figure 2 below represents the presence of signal peptide regions in the candidate glycoprotein for vaccine design.

The glycoprotein sequence of 1434 amino acids was submitted for prediction using the ProtParam prediction server. The prediction showed that the glycoprotein has a molecular weight of 162.19 kDa and an isoelectric point of 7.68. The instability index of the glycoprotein is 39.86, indicating stability, as the index is less than 40. The GRAVY score of the glycoprotein is −0.099, indicating a hydrophilic nature, which is consistent with the glycoprotein being a surface-attached viral attachment protein. Table 1 below represents the physicochemical properties of the candidate glycoprotein for vaccine design.

The top sequence similarities identified by BLASTp were with Batai virus and related orthobunyavirus proteins. The top-ranked Batai virus polyprotein had 100% query coverage and 95.61% identity with an E-value of 0.0, with subsequent high-ranking matches being related glycoproteins/polyproteins from different viruses. The top BLASTp hits did not contain any human protein; thus, no clear high-confidence human homolog was found in the results that were explored.

### 2.3. Identification and Selection of B-Cell and T-Cell Epitopes

B-cell linear epitope prediction results were used to identify specific areas of the BATV glycoprotein where antibody response could be elicited. The results of the B-cell linear epitope prediction were as follows: the average score for the prediction was 0.469, and the range of the score varied from 0.156 to 0.672. Of the 51 peptides predicted, the top 5 were selected for their length, antigenicity, and hydrophilicity (Table 2). The epitopes of particular interest were the strong continuous B-cell linear epitopes with specific properties.

**Figure 3:**
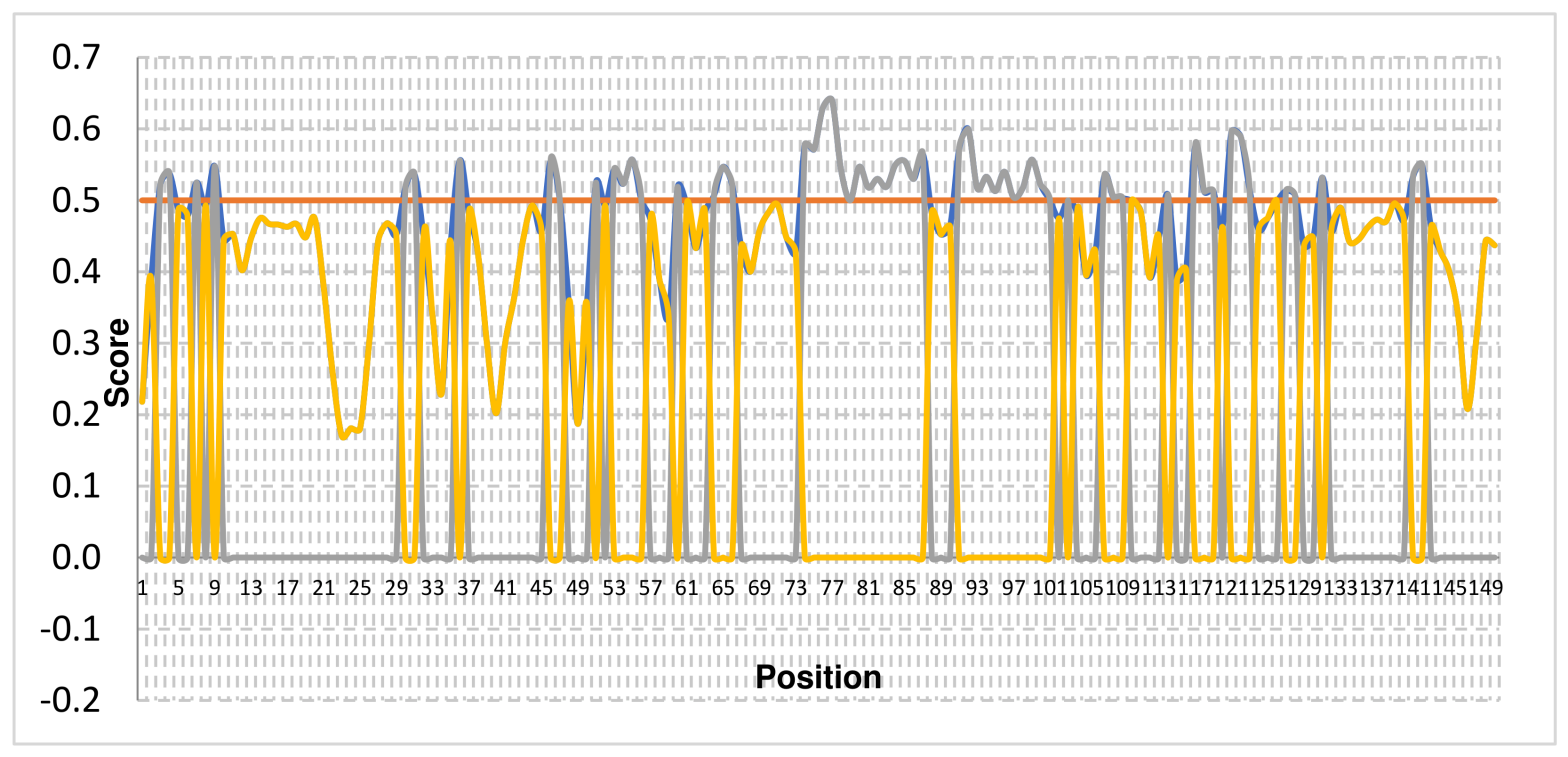
The division and positioning of B-cell epitopes in the selected glycoprotein for the vaccine construction. The gray line represents the prediction score profile, the orange line indicates the predicted B-cell epitope profile, and the red horizontal line represents the threshold value (0.5) used for epitope prediction.

Figure 3 below represents the division of B-cell epitopes within the candidate glycoprotein utilized for vaccine construction.

In order to induce a cellular response, we screened the glycoprotein for CTL epitopes. From the screening, 17 high-binding 9-mer peptides emerged as significant (as shown in Table 3). Among these, epitopes like MPLFMPIAY and ALFLLMALV were prioritized due to their potential ability to interact with a wide range of MHC Class I alleles, thus enhancing the chances of successful clearance of infected cells.

Helper T Lymphocyte (HTL) epitopes are important in regulating the immune response and creating memory. A total of ten 15-mer HTL epitopes were identified (refer to Table 4). From these epitopes, CQGYKSLRVARLLCK and SLEEFKRAISEKLSH were selected for their high binding capacity to MHC Class II molecules and their potential to elicit crucial cytokines such as Interferon-gamma (IFN-γ).

### 2.4. Design and Characterization of the Multi-Epitope Vaccine (MEV)

**Figure 4:**
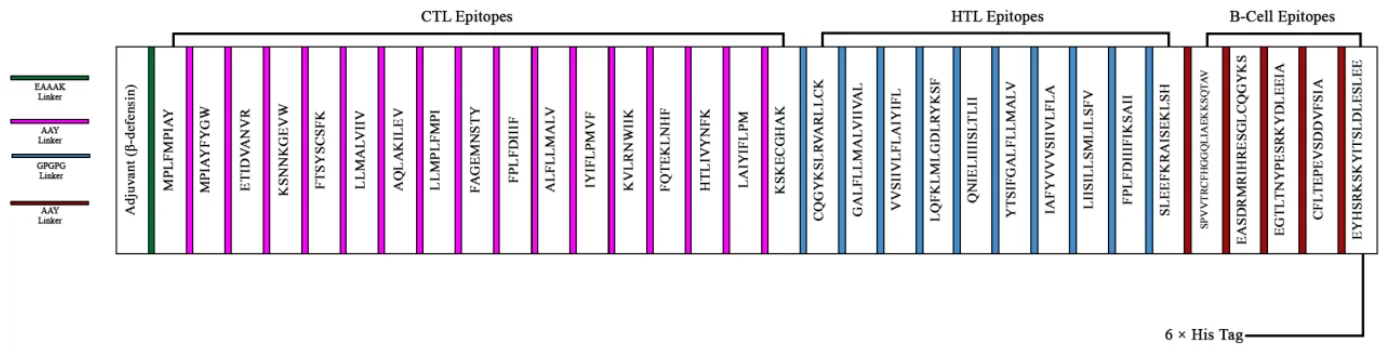
The construct map of the designed vaccine chimera indicating the merging of B-cell, CTL, and HTL Epitopes with suitable linkers, adjuvants, and a 6×His Tag.

The chimeric MEV was designed by stringing together the best B-cell, CTL, and HTL epitopes in one peptide. To further increase innate and adaptive immunity, a beta-defensin sequence of 45 amino acids was incorporated as an adjuvant at the N-terminus of the chimeric MEV. The choice of human β-defensin as an adjuvant was based on previous reports of its immunomodulatory activity and its role in the induction of innate and adaptive immune responses. The incorporation of it was intended to build up the predicted immunogenic potential of the construct, but its activity and safety in the current vaccine formulation has yet to be experimented [10]. The adjuvant was then covalently attached to the epitope regions using a rigid spacer of EAAAK to maintain the conformation of the individual peptides. The CTL epitopes were then covalently linked using AAY linkers, which are beneficial in proteasomal processing and MHC-I-mediated antigen presentation. The HTL and B-cell epitope regions were then covalently linked using GPGPG and KK linkers, respectively. The GPGPG linker is beneficial in inhibiting the formation of new epitopes and stimulating helper T-cell activation. The KK linker is beneficial in maintaining the immunogenicity of the B-cell epitopes. A 6×His tag was incorporated at the C-terminus of the chimeric MEV for purification and identification purposes (Supplementary Table S4). Figure 4 below represents the construct map of the designed vaccine chimera.

### 2.5. Vaccine Antigenicity and Allergenicity Evaluation

The antigenic potential of the multi-epitope construct was verified using the Vaxijen v2.0 tool, with the virus option corresponding to the expression system. The vaccine has an antigenic score of 0.7959, which can be considered a protective antigen. There has been a significant rise in the antigenic score compared to the native glycoprotein. This rise indicates that the designed construct with high CTL, HTL, and B-cell epitopes, along with the adjuvant activity of the β-defensin, results in a construct with increased predicted antigenicity. Safety is critical for chimeric constructs. To determine the safety profile of the designed construct, AllerTOP v2.1 was used to analyze for the presence of allergens that may result from the fusion of the epitopes with the linker regions. The analysis resulted in the vaccine construct being classified as a Probable Non-Allergen. The similarity with the closest sequence in the database corresponds to the calcium channel protein from *Oryza sativa*, which confirms that there are no allergenic motifs associated with hypersensitivity in humans.

### 2.6. Vaccine Structure Prediction and Validation

**Figure 5:**
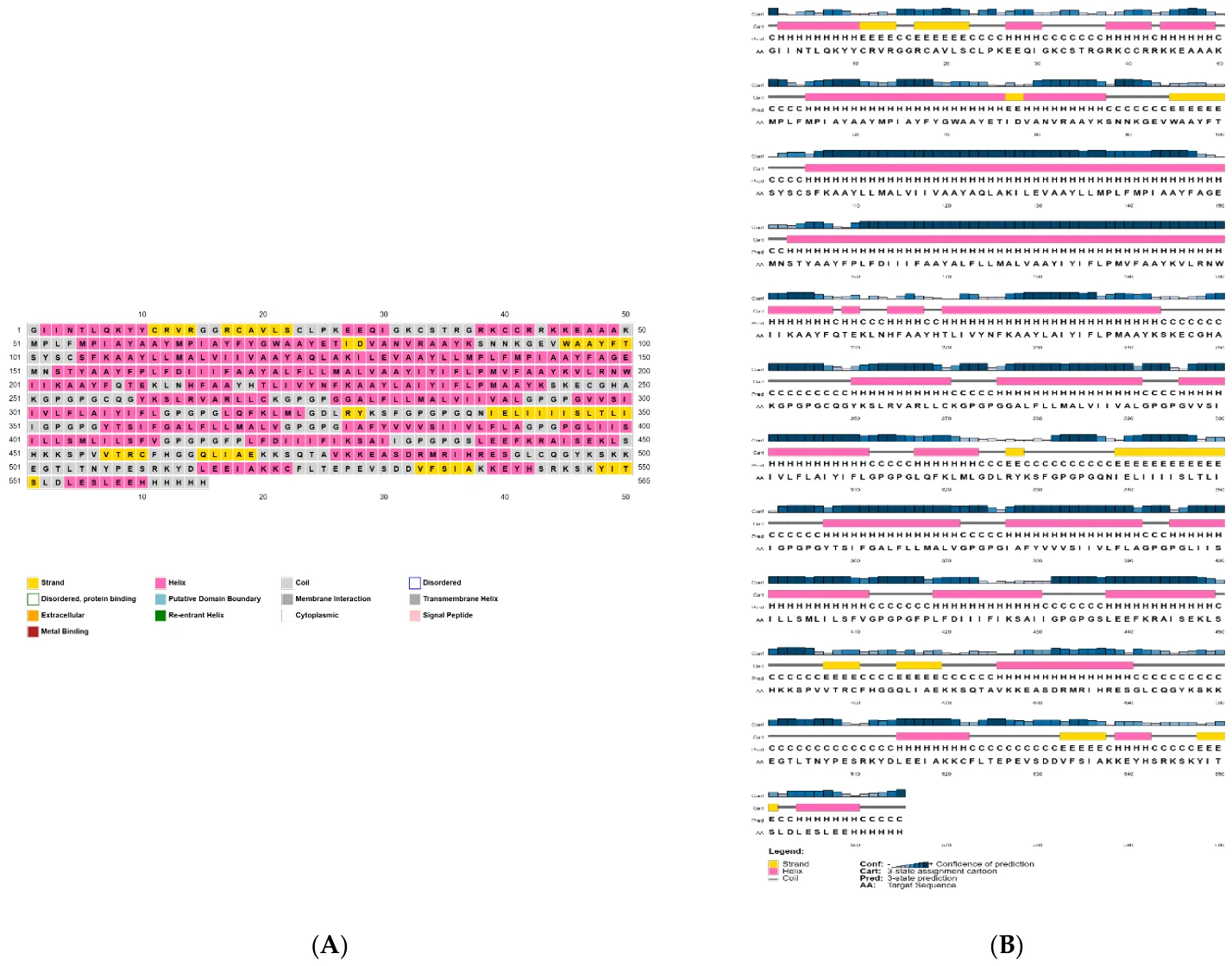
Secondary structure prediction of the designed vaccine construct: (**A**) Annotation Grid for the predicted secondary structure; (**B**) PsiPred Cartoon of the predicted secondary structure.

In order to understand how the proposed multi-epitope vaccine based on BATV folds, its secondary structure prediction was carried out. The 565 amino acids in the vaccine are composed of alpha-helices, extended strands, and random coils. It is worth noting that the presence of coiled structures indicates the level of flexibility required for effective epitope display. The secondary structure of the designed vaccine chimera is given below in Figure 5.

**Figure 6:**
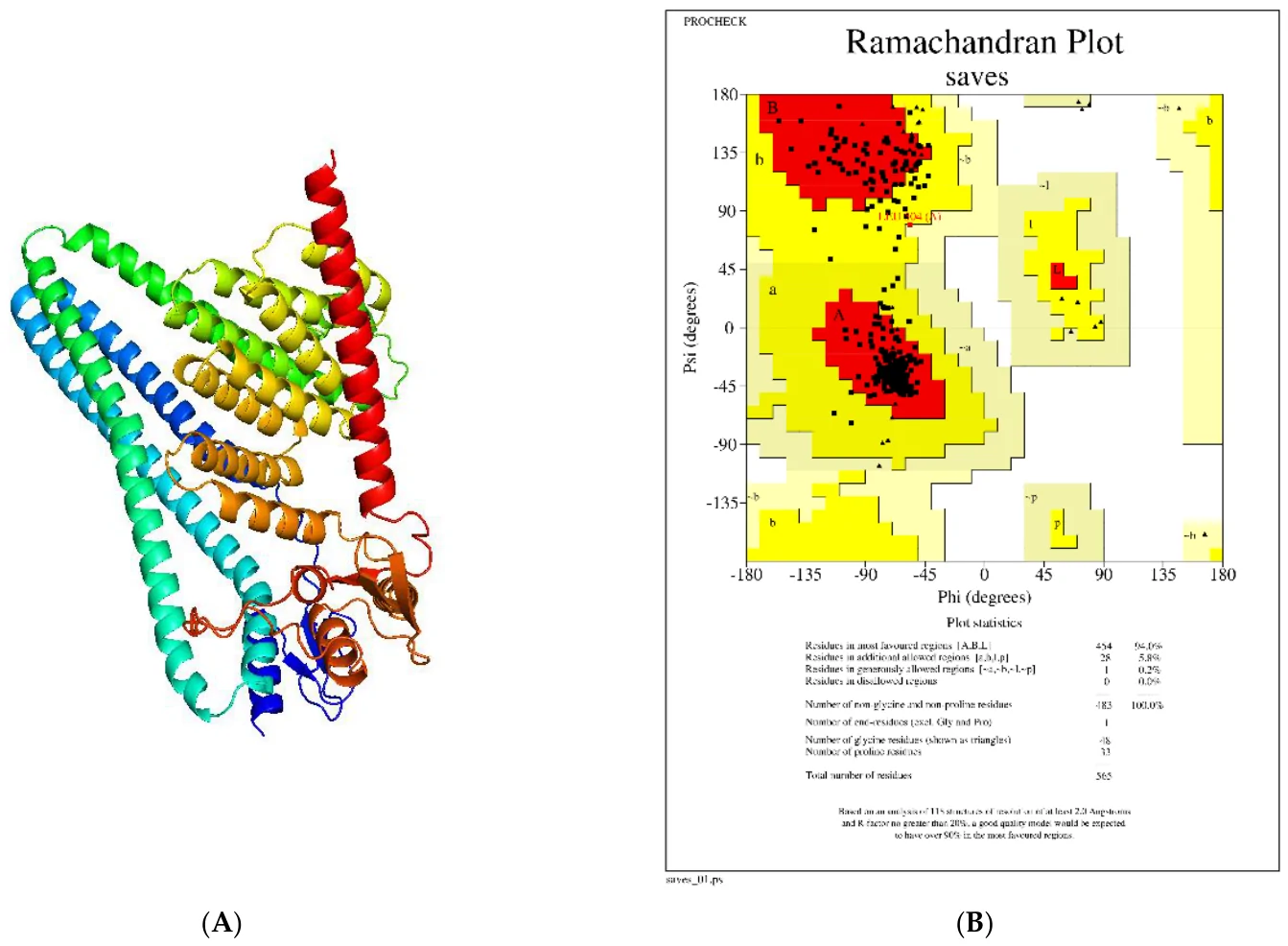
Structure prediction and validation of the vaccine structure. (**A**) The predicted tertiary structure of the designed vaccine chimera indicating the loops, helices, and beta strands in the structure. (**B**) The Ramachandran plot assessing the quality of the vaccine structure.

A three-dimensional model of the vaccine construct was generated using AlphaFold and subsequently assessed for stereochemical quality. The initial prediction of the 3D structure was refined to ensure chemical and geometric accuracy. The final structure was compact and stable, effectively combining the adjuvant, linkers, and epitope domains in one functional unit. The 3D structure of the vaccine was validated and analyzed rigorously using the SAVES v6.1 (PROCHECK) server. The Ramachandran plot indicates that 94.0% of the residues are in the most favored regions, 5.8% are in the additionally allowed regions, 0.2% are in the generously allowed regions, and 0.0% are in the disallowed regions. The structure is thus highly reliable, as it exceeds the standard level of 90%. AlphaFold confidence assessment (pTM) was 0.16, which suggested low confidence in the global structure of the multi-epitope construct. Residue-level confidence was determined from the pLDDT profile, and the lower-confidence regions were read with caution, especially in the flexible regions of the linker. Hence, it was decided that the predicted structure would be regarded as a computational structural model and not as a representation of the native structure. The predicted tertiary structure of the vaccine construct is given below in Figure 6A, and the predicted Ramachandran plot is given below in Figure 6B.

### 2.7. Molecular Docking of the Vaccine Construct with Human TLR4 Receptor

To assess whether the multi-epitope vaccine has the ability to trigger an innate immune response, the vaccine construct was docked against the human TLR4 receptor using the ClusPro 2.0 server. TLR4 is the receptor that recognizes the β-defensin adjuvant and initiates the inflammatory response that is required for an effective vaccine response. The docking results revealed many possible docked conformations, and these were grouped into clusters based on representative energy scores. The complex of particular interest was Cluster 0, which comprised 45 docked conformations, suggesting strong convergence of the sampling method. This cluster had a representative weighted score of −1194.8, and the lowest energy in this cluster was as high as −1325.5.

**Figure 7:**
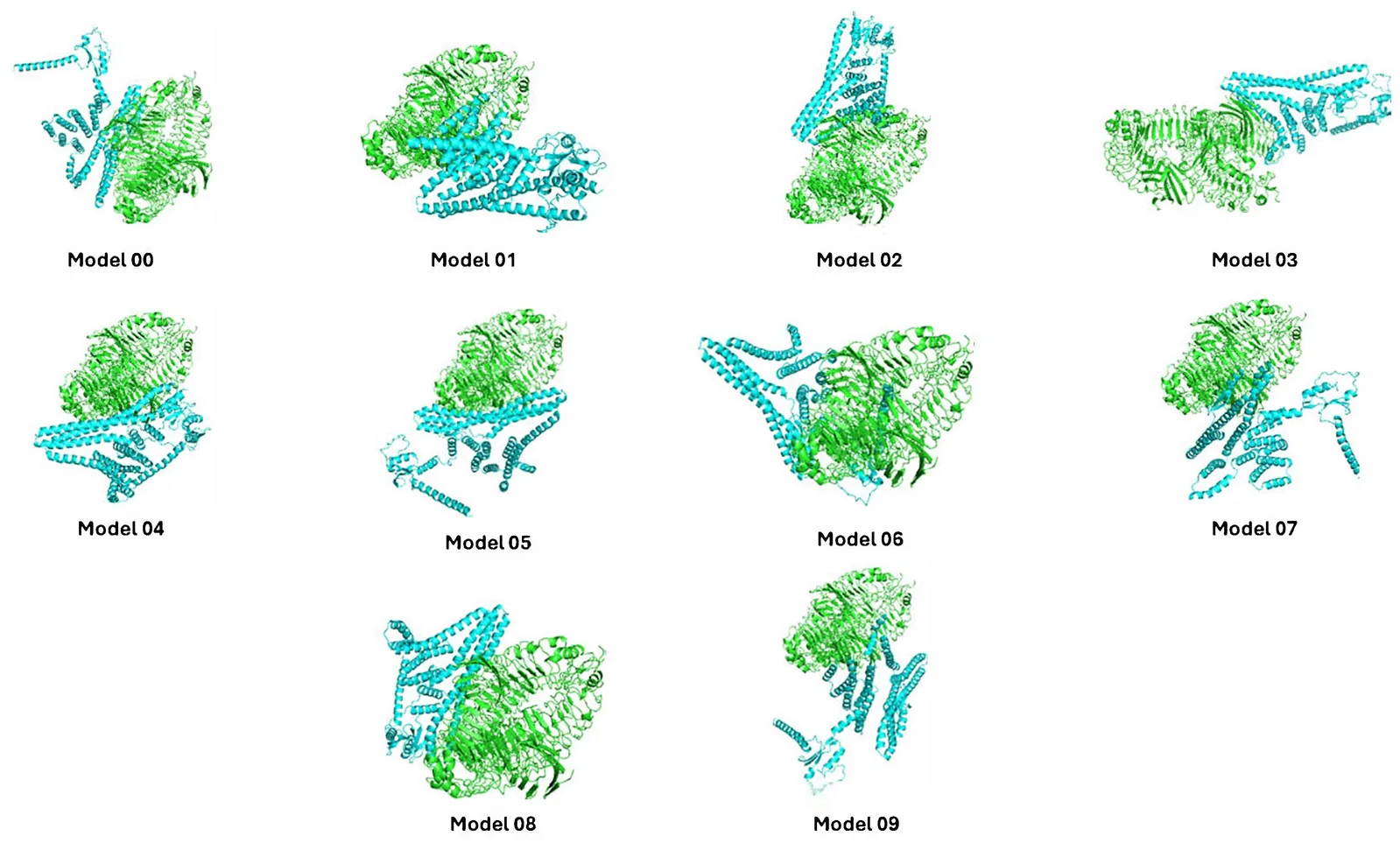
The top 10 complexes of the designed vaccine chimera and human TLR4 receptor docking indicating the strong binding of the vaccine to the human TLR4 receptor. The green color represents the receptor while the blue color represents the vaccine construct.

The highest-ranking docking cluster had a representative ClusPro weighted score of −1194.8. Note that this is a docking ranking score that is dimensionless and was calculated by the ClusPro scoring function, not an experimentally measured binding free energy. The more negative the weighted score, the more favorable the docking conformation is in the ClusPro framework. The negative ClusPro weighted scores indicated relatively favorable predicted docking poses within the scoring framework. However, these scores do not constitute experimental binding affinities or direct evidence of downstream TLR4 signaling. Figure 7 below represents the top 10 docking complexes of the molecular docking with the human TLR4 receptor.

### 2.8. Interaction Profiling

To identify the precise molecular contacts that facilitate the retention of the vaccine at the TLR4 site, the best docking pose is analyzed with PyMOL v2.5.2. Upon analysis, the interaction interface is identified with a 4 Å cutoff, where 193 atoms are found at the interaction site. Detailed analysis of the interaction site reveals the presence of many polar contacts and H-bonds, as depicted in the figure below. Key residues that facilitate the interaction between the human TLR4 and the vaccine adjuvant include Arg67, Arg87, Lys47, Glu42, and Asn44. Table 5 below represents the detailed interaction profile of the human TLR4 receptor residues with the BATV vaccine construct.

**Figure 8:**
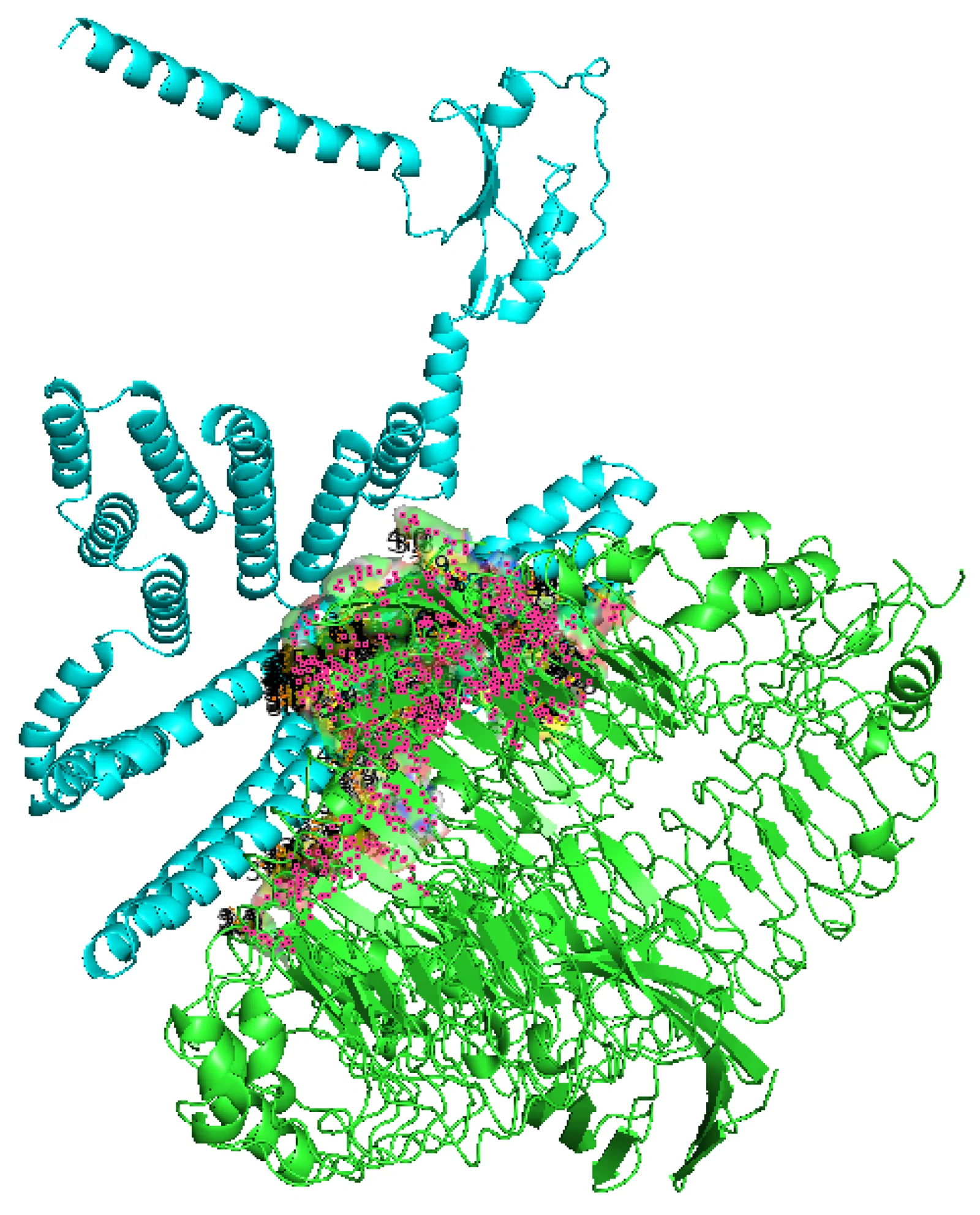
Interaction profiling of the vaccine and human TLR4 receptor docking complex indicating the strong binding affinity and molecular contacts within the complex.

The presence of polar amino acid residues at the interaction site reveals that electrostatic complementarity is essential in the interaction between the vaccine adjuvant and the epitopes at the active site of the TLR4 receptor. Besides the polar contacts, the interaction site is characterized by the presence of hydrophobic amino acid residues, including Pro28, Leu43, Ile48, and Phe63. These residues, which facilitate van der Waals contacts, are highlighted as orange spheres. A transparency-mapped surface reveals that the ligand is well accommodated at the site. Figure 8 below represents the molecular contacts in the vaccine and human TLR4 receptor docking complex.

### 2.9. Population Coverage Analysis

**Figure 9:**
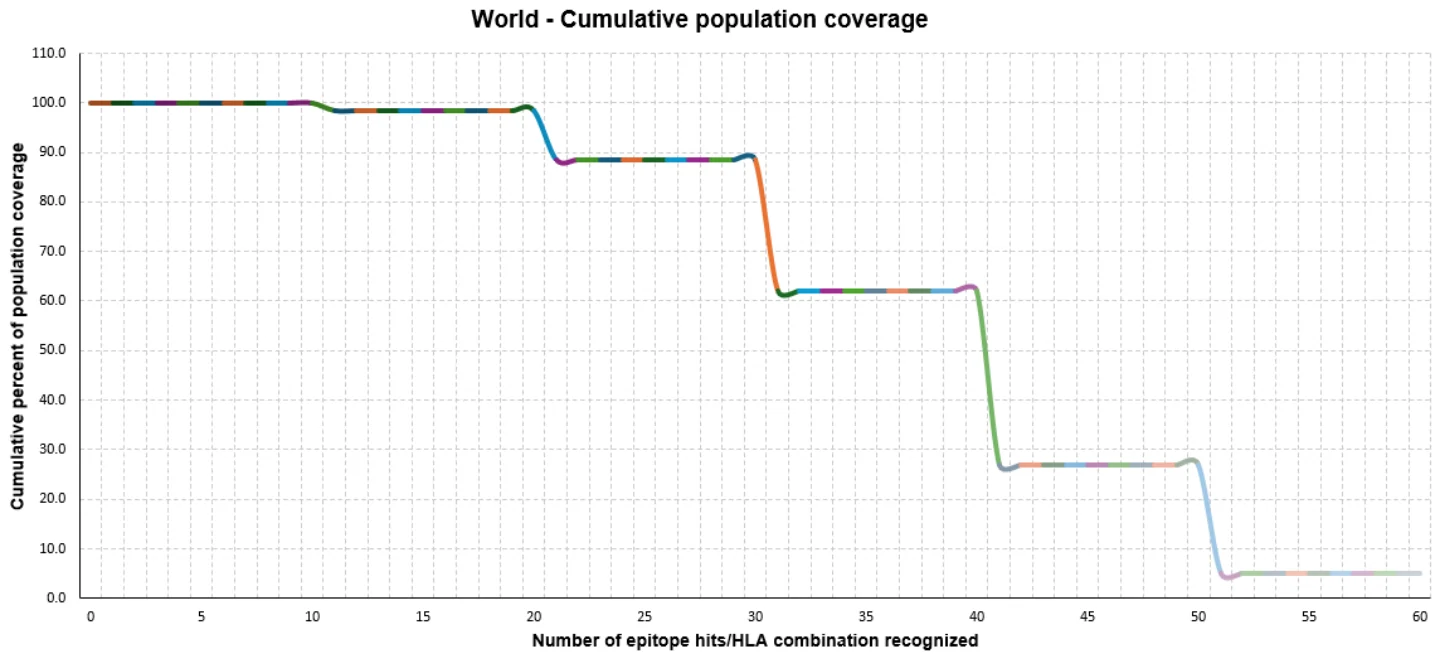
World-class combined population coverage of the designed vaccine construct indicating the number of epitope hits and percentage of individuals.

A universal vaccine has to be able to cover the globe with a unique mix of human populations, each with their unique HLA allele types. In the case of the BATV vaccine, we used the IEDB Population Coverage tool (https://tools.iedb.org/population/) to evaluate the combined MHC Class I and II epitopes. The cumulative percentage coverage was found to be 99.92%, indicating that the chosen chimeric epitopes have the capability of interacting with the HLA alleles of almost the a broad proportion of the modeled global population. The average hit rate, which is the average number of epitope–HLA combinations that any individual can recognize, is quite high at 38.07. The PC90 is the minimum number of epitope hits that any individual is able to recognize. In this case, the PC90 is 28.52. In essence, this means that the least represented 10 percent of the population is still able to recognize more than 28 unique epitope–HLA combinations from this vaccine. Figure 9 below represents the world-class combined population coverage of the designed vaccine construct, indicating the number of epitope hits and percentage of individuals. The predicted population coverage was 99.92%, with an average of 38.07 epitope–HLA combinations predicted per person. The value of the reported PC90, 28.52, represents the minimum number of epitope–HLA combinations that can be recognized by 90% of the modeled population. These figures are global averages and may not be seen as equal coverage at the level of geographic and ethnic populations.

### 2.10. Normal Mode Analysis

**Figure 10:**
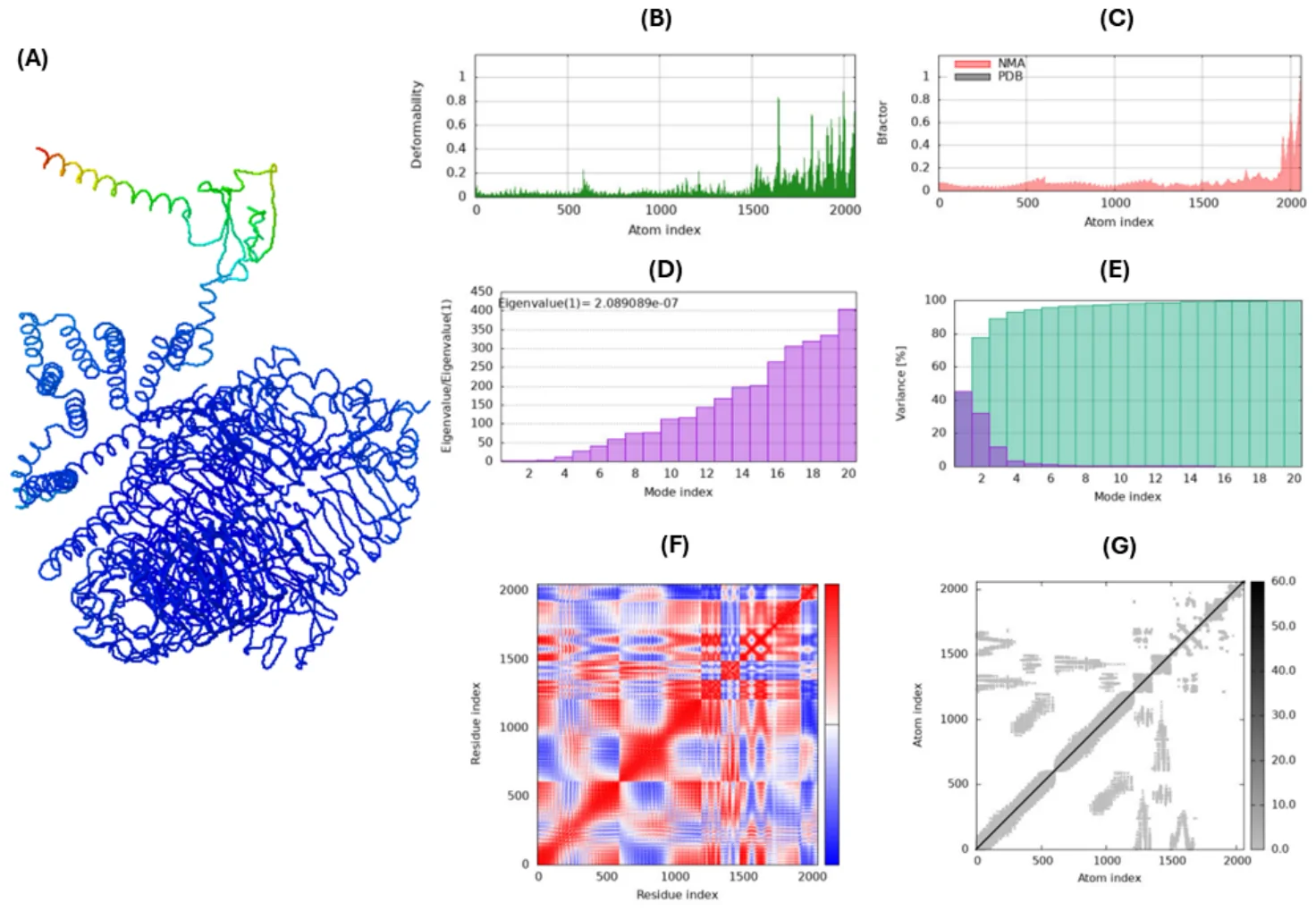
Normal Mode Analysis (NMA) of the BATV multi-epitope vaccine–TLR4 complex. (**A**) 3D representation of the docked complex showing the collective motions (NMA-derived B-factors); (**B**) deformability plot illustrating the potential hinges and flexible regions of the structure; (**C**) comparison of B-factor values calculated by NMA (red) and the PDB average (gray); (**D**) eigenvalue of the complex, indicating the energy required for structural deformation; (**E**) variance associated with each mode (purple) and the cumulative variance (green); (**F**) covariance matrix showing correlated (red), anti-correlated (blue), and uncorrelated (white) motions between residue pairs; (**G**) elastic network model representing the stiffness of atom–atom connections (gray dots) and the stiffness matrix (black diagonal).

For the analysis of collective motions, flexibility, and dynamic stability of the BATV multi-epitope vaccine–TLR4 complex, Normal Mode Analysis (NMA) was carried out on the iMODS server. Figure 10A shows the overall structure of the docked complex and the overall motion pattern. The deformability profile was generally low throughout the complex, with some peaks in the deformability occurring in the more flexible areas of the structure (Figure 10B). These local fluctuations are in line with the flexibility of both loop and linker regions of proteins that can allow for conformational change and exposure of epitopes without compromising the overall structure of the receptor-bound complex. The B-factor profile derived from the NMA also suggested relatively restricted mobility throughout the majority of the complex with increased mobility in particular flexible regions (Figure 10C).

The complex had an eigenvalue of 2.08 × 10^−7^ (Figure 10D), which is consistent with the energy needed to deform along the normal mode that was analyzed and confirmed the presence of collective low-frequency motions within the complex. The variance plot showed that the first normal modes had more significant contributions to the conformational motion, while the cumulative variance increased in successive modes (Figure 10E). The covariance matrix showed high regions of positive correlation between the motions (represented in red), indicating coordinated motions between various regions of the vaccine–TLR4 complex, whereas regions of negative correlation (represented in blue) showed anti-correlated motions where the structural regions were moving in opposite directions (Figure 10F). Correlated and localized anti-correlated motions indicate global coordinated motion of the complex, yet localized conformational changes are still possible, especially in the flexible parts of the complex (linker and epitope-associated). Finally, the elastic network map showed that the stiffness of the inter-residue connections was not uniform across the entire complex, with some regions having flexibility (Figure 10G), which is consistent with the presence of both structurally constrained regions and localized flexibility. The overall architecture of the vaccine–TLR4 complex is dynamically stable, as revealed by the NMA results, but it is still flexible in some regions, a property that might allow for conformational adaptation to the TLR4 receptor during recognition.

### 2.11. In Silico Immune Simulation

**Figure 11:**
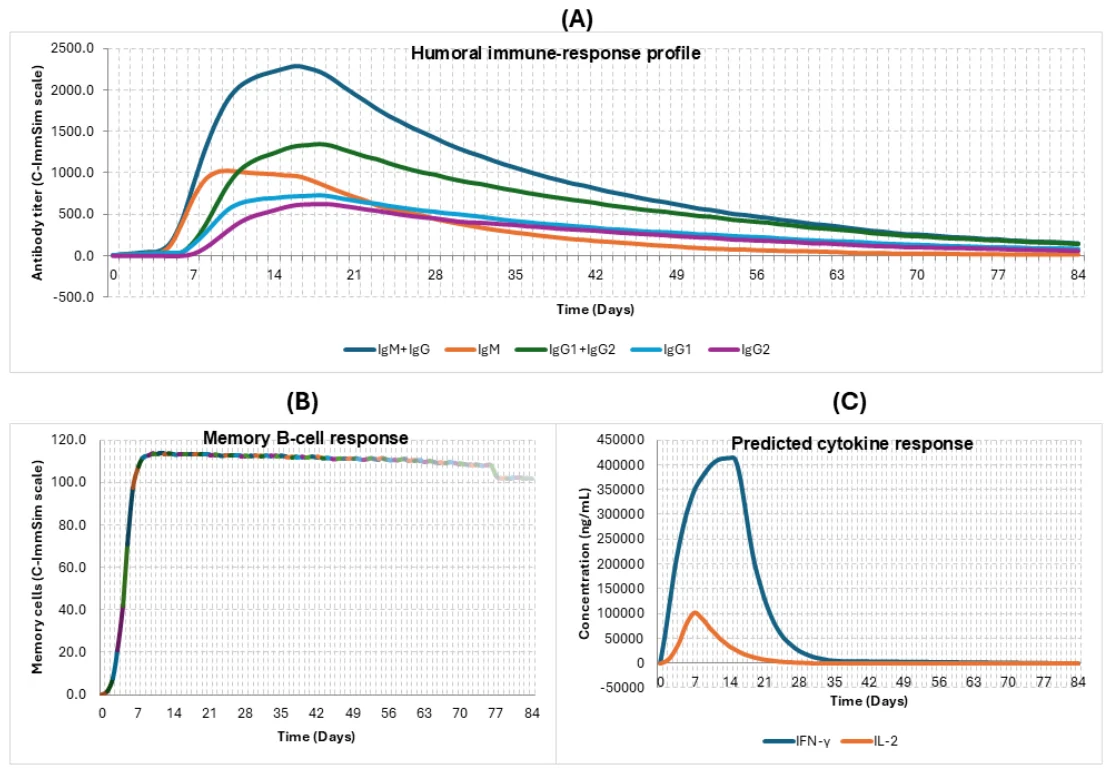
C-ImmSim-predicted immune responses of the multi-epitope vaccine over 84 days: (**A**) humoral antibody response, (**B**) memory B-cell response, and (**C**) IFN-γ and IL-2 cytokine profiles.

C-ImmSim predicted that the kinetics of the immune response showed distinct humoral and cellular immune response on 84 days of simulation (Figure 11). C-ImmSim was simulated using the volume 10 and random seed 12,345. Vaccine construct was administered as outlined in the simulation schedule and the simulation was run for 84 days to allow for evaluation at weeks 1, 4, 8, and 12. Humoral response demonstrated early IgM response, along with higher IgG1 and IgG2 responses (Figure 11A). At weeks 1, 4, 8, and 12, the approximate IgM titers were 696.3, 439.5, 60.9, and 7.6, respectively, whereas the corresponding combined IgG1 + IgG2 titers were 159.8, 977.9, 405.3, and 137.0. Memory B-cells were well-maintained during the simulation with approximate counts of 107.4, 112.9, 110.5, and 102.3 at week 1, 4, 8, and 12, respectively (Figure 11B). Predicted response was strong, with the cytokine level of IFN-γ reaching ~351,370 ng/mL at week 1 and decreasing over time to ~24,658 ng/mL at week 4, and to near-baseline levels at later timepoints (Figure 11C). The overall simulation model predicted an early cytokine response, followed by sustained antibody and memory B-cell profiles, but the values presented are model-based predictions of immune responses, not experimentally measured values. Table 6 below represents the timepoints of immune simulations at the 12 week interval.

Figure 11 below represents the results of immune simulation analysis.

### 2.12. Reverse Translation and Codon Adaptation

The multi-epitope vaccine construct consisting of 565-amino-acid sequences was successfully reverse translated to a coding sequence of 1695 bp using Sequence Manipulation Suite. This nucleotide sequence was then optimized for expression in *E. coli*. K-12 strain using the VectorBuilder codon optimization tool. The optimized and initial sequences had a GC content of 51.67% and 52.49%, respectively, and a codon adaptation index (CAI) of 0.93 and 1.00, respectively. The optimized sequence was thus designed with a high CAI and a balanced GC content, which is thought to be suitable for the *E. coli* K-12 expression system. The results serve as computational support for the following steps of recombinant cloning and experimental expression of the designed vaccine construct.

## 3. Discussion

The purpose of the current study is to determine if a computational method might be able to overcome the obstacles facing traditional vaccinology to develop a viable defense mechanism for the Batai orthobunyavirus [11]. Through an exhaustive analysis of the BATV Envelopment Glycoprotein, a chimeric vaccine was created that not only possesses a high antigenic potential, but one which avoids the allergenic and autoimmune side effects normally associated with whole-virus vaccines. The greatest advantage of this method has been its incredible adaptability, as the results suggest that the epitopes selected are capable of varying across a wide range of human genetic backgrounds, thus providing a defense mechanism for a vast percentage of the world’s population [12]. Furthermore, the high-affinity binding properties of the docking simulation with the TLR4 receptor demonstrate that the vaccine has a structural configuration that activates the innate immune system from the onset, providing a strong, stable, and immunogenic platform for answering the research question [13].

The significant improvement in antigenicity from the native glycoprotein to the final construct, reaching a score of 0.7959, demonstrates that the strategy of concentrating high-affinity epitopes while filtering out non-essential viral regions successfully amplified the immune signal [9]. This shift suggests that the vaccine functions as a potent immunogen specifically engineered for enhanced recognition by the host’s immune system. Furthermore, the molecular docking and stability data confirm a thermodynamically favorable interaction with the TLR4 receptor, indicating that the construct is structurally primed to trigger the innate inflammatory pathways necessary for a robust, long-lasting protective response [8].

In the context of recent immunoinformatics studies, the present vaccine construct can be evaluated. A study on the development of a multi-epitope vaccine for Batai orthobunyavirus (Bunyas) for targeting both the nucleoprotein and the envelope polyprotein was reported with a combined global population coverage of 98.62%, and favorable prediction results of antigenicity, structural stability, docking with TLRs, molecular dynamics, and immune simulation [10]. The BATV envelope glycoprotein was selected for the present study, and a slightly higher predicted global population coverage (99.92%) was obtained, indicating good coverage of HLA among the selected epitopes. Likewise, a 2026 multi-epitope vaccine design for Oropouche orthobunyavirus (OoBV) was developed based on the envelopment polyprotein, achieving 94.07% coverage of the global population and demonstrating positive docking properties with TLR4, favorable molecular dynamics, and simulated immune response. While human β-defensin was chosen due to its immunomodulatory properties, other adjuvants such as other TLR-targeting immunostimulatory molecules are also investigated in the design of multi-epitope vaccines. Effectiveness and safety of these strategies may be antigen or delivery system dependent. Thus, the predicted immunostimulatory activity, possible toxicity and receptor-mediated activity of β-defensin in the existing BATV construct should be taken with a pinch of salt until proven experimentally. These studies all argue in favor of envelope-associated proteins being good vaccine candidates for orthobunyavirus [14]. Due to the antigen selection, epitope composition, HLA allele diversity, adjuvants, structural modeling, and population datasets, population coverage percentages and docking scores should be considered in relation to one another and not as absolute tools for vaccine efficacy. The 99.92% population coverage reported in the current studies is therefore a good computational prediction, which will need experimental verification [15].

Although the immunoinformatics data appears to be very promising, we should remain mindful of the following constraints that have been built into the process. First and foremost, the key constraint is that this data is based on computational tools and predictive models. While the tools used, such as AlphaFold and C-IMMSIM, have access to vast amounts of data, they still cannot replicate the actual biology of the living organism. In addition, the individual host organism’s biology, the post-translational modifications, and the actual cell biology of the protein folding process may influence the effectiveness of the vaccine. Furthermore, the actual stability of the vaccine and the actual binding affinity to the human TLR-4 receptor still have to be validated with actual experimentation. While the docking analysis showed good predicted docking interactions with TLR4, the ClusPro weighted scores are not thermodynamic binding energies but rank docking scores. Thus, the results have to be read as evidence for receptor recognition but not necessarily for binding affinity. Comparative docking with experimentally known complexes (e.g., the complex of human TLR4–MD2–LPS) in combination with molecular dynamics simulations and binding free energy calculations (e.g., MM/PBSA or MM/GBSA) should be performed to further validate the stability and biological relevance of the predicted interaction in future studies [16,17].

The construct represents a computationally prioritized candidate with broad predicted HLA population coverage [18]. The ability to cover the global population with 99.92% efficacy demonstrates the possibility of a “universal” vaccine, effective in the face of genetic heterogeneity, a crucial consideration in biosecurity and pandemic preparedness. From a practical perspective, the 6×His tag and the stability of the chimeric protein may facilitate subsequent recombinant expression studies in various systems such as *E. coli*. This computational method not only addresses the current BATV concern but may serve as a template for the rapid development of vaccines for other emerging or neglected orthobunyaviruses. A population-specific breakdown by geographic or ethnic group was not performed; therefore, potential under-representation of particular HLA backgrounds cannot be excluded and should be examined in future population-stratified analyses [19].

In order to go beyond computer modeling and prove things in the real world, the next step will be to transition to wet lab validation. In this area, the focus will be on constructing the chimeric gene to express it in an appropriate host organism, in order to observe how the protein actually folds and if it remains soluble [20]. The validation should be carried out in a step-wise experimental process in the future. The codon optimized vaccine gene should be first be cloned into an appropriate expression vector suitable for bacteria such as *E. coli* K-12, expressed in the bacteria, purified by the C-terminal 6×His tag, and the protein identity and solubility should be confirmed. It is then important to assess the functional activity of the construct in vitro, to see if it is capable of inducing receptor associated signaling, by using TLR4 responsive reporter or cytokine assays. Antigen-specific cellular immunity should be evaluated by human or animal lymphocytes by IFN-γ/IL-2 ELISpot and/or by intracellular cytokine staining assays, in addition to antibody-response assays. Then, constructs with acceptable expression, stability, and immunogenicity should be tested in an appropriate small animal model for safety, immunogenicity, persistence of immune memory, and (if a BATV challenge model exists) protection against a viral challenge.

## 4. Materials and Methods

### 4.1. Proteome Retrieval and Candidate Selection

The complete proteome of Batai orthobunyavirus (BATV) was downloaded from the NCBI database (https://www.ncbi.nlm.nih.gov) (NCBI Accession: GCA_004789555.1) (accessed on 9 March 2026). To develop an efficient chimeric multi-epitope vaccine, the proteome was scanned for proteins that have high antigenic potential and accessibility. From the structural proteins, Envelop Glycoprotein (Accession: UZT55202.1) was chosen as the first vaccine candidate. This glycoprotein, being the major surface component of the virus, plays an important role in the attachment and entry of the virus [21].

### 4.2. Evaluation of Antigenicity and Allergenicity

The amino acid sequence of the BATV glycoprotein was examined for its ability to induce a strong immune response and its safety, using specific bioinformatics software. To check for the antigenic properties of the glycoprotein, the VaxiJen v2.0 server (https://www.ddg-pharmfac.net/vaxijen/VaxiJen/VaxiJen.html) (accessed on 9 March 2026) [22], using a virus-specific model and a 0.4 threshold, was used to check whether the glycoprotein sequence could function as a protective antigen. At the same time, the ability of the candidate to induce allergic responses was checked using the AllerTOP v2.1 server (https://www.ddg-pharmfac.net/allertop_test), (accessed on 9 March 2026) which uses an alignment-free method to check the properties of the amino acid sequence and determine whether the glycoprotein sequence could be classified as allergenic or non-allergenic. This two-pronged approach ensured the glycoprotein selected as the candidate for the multi-epitope vaccine was both highly immunogenic and safe [23,24].

### 4.3. Physiochemical Characterization & Topology Prediction

The BATV glycoprotein was further analyzed to confirm its viability and its accessibility on the surface by determining its structure and chemical properties. To determine the location of the BATV glycoprotein within the membrane and which part of the protein is on the outside, TMHMM v2.0 was used to determine its topology (https://services.healthtech.dtu.dk/services/TMHMM-2.0) (accessed on 10 March 2026) [25]. Additionally, the presence or absence of the signal peptide on the protein’s N-terminus was determined by SignalP 6.0 (https://services.healthtech.dtu.dk/services/SignalP-6.0) (Accessed on 10 March 2026) [26], which identified the cleavage site. ExPASy ProtParam (https://web.expasy.org/protparam) (accessed on 10 March 2026) determined the chemical properties of the BATV glycoprotein, such as its molecular weight, theoretical pI, instability index, and GRAVY [27].

### 4.4. Homology Search and Autoimmunity Screening

The homology of sequences was analyzed by the NCBI bioinformatics program BLASTp (https://blast.ncbi.nlm.nih.gov/Blast.cgi) (accessed on 11 March 2026) with the BLOSUM62 substitution matrix and E-value threshold of 0.05. The Batai virus glycoprotein (UZT55202.1; 1434 amino acids) was used as a query and the sequence identity, query coverage, and E-values of the resulting alignments were investigated [24].

### 4.5. T-Cell and B-Cell Epitope Mapping

#### 4.5.1. Linear B-Cell Epitope Prediction

Linear B-cell epitopes were predicted using the IEDB B-cell prediction platform (https://tools.iedb.org/main/bcell) (accessed on 12 March 2026) using the method of Bepipred linear epitope prediction. Lead peptides were selected based on the high prediction score, hydrophilicity, and high degree of surface accessibility (Supplementary Table S1) [28].

#### 4.5.2. MHC Class I (CTL) Epitope Prediction

To identify the Cytotoxic T Lymphocyte (CTL) epitopes, the glycoprotein sequence was used as input to the IEDB MHC-I server (https://tools.iedb.org/mhci) (accessed on 12 March 2026). Peptides of length 9 were identified as CTL epitopes based on high affinity, with the IC50 threshold set to less than 10 nM. Peptides with high affinity bind to a wide range of HLA molecules, thereby facilitating the destruction of virus-infected target cells. The selection did not rely on percentile ranks and separate CTL immunogenicity scores as primary criteria in the present workflow, but on the predicted MHC-I binding affinity and later on the antigenicity and safety assessment (Supplementary Table S2) [29].

#### 4.5.3. MHC Class II (HTL) Epitope Prediction

To identify the Helper T Lymphocyte (HTL) epitopes, the prediction of the 15-mer peptides with high affinity to the MHC Class II molecules was conducted using the IEDB MHC Class II epitope prediction tool (https://tools.iedb.org/mhcii) (accessed on 12 March 2026). Peptides were identified based on their high affinity, with special consideration of their ability to induce key cytokines, particularly Interferon-gamma (IFN-γ), which plays a key role in the coordination of long-term immunological memory. The fifteenmer HTL epitope predictions were generated with the IEDB MHC class II prediction tool and were screened primarily for their strong predicted MHC-II binding affinity (IC_50_ < 10 nM). Peptides that bound to more than one HLA class II molecule were prioritized. Prediction information was used qualitatively for cytokine-inducing potential, and quantitative scores for IFN-γ induction were not used as a necessary requirement for all HTL epitopes (Supplementary Table S3) [30].

### 4.6. Chimeric Multi-Epitope Vaccine Construction

The final chimeric vaccine was constructed by combining the top B-cell, CTL, and HTL epitopes in one peptide chain. The immunogenicity of the chimeric vaccine was enhanced by inserting 45 amino acids of beta-defensin at the N-terminus as an adjuvant. Appropriate linkers were incorporated to facilitate epitope separation and antigen processing. The adjuvant and the chimeric peptide are connected by the rigid EAAAK spacer, and the CTL, HTL, and B-cell segments are connected by AAY, GPGPG, and KK spacers, respectively. The 6×His tag is appended at the C-terminus for potential downstream purification and characterization (Supplementary Table S4) [31].

### 4.7. Structural Modeling and Validation

#### 4.7.1. Secondary Structure Prediction

The secondary structure composition, that is, alpha-helices, extended strands, and random coils, of the vaccine was predicted using PSIPRED (https://bioinf.cs.ucl.ac.uk/psipred) (accessed on 13 March 2026). These prediction programs were used to estimate the level of flexibility required for effective epitope display [32].

#### 4.7.2. Tertiary Structure Prediction and Refinement

The 3D structure of the vaccine was constructed using AlphaFold3 (https://alphafold.ebi.ac.uk), based on deep learning algorithms that generate high-confidence 3D structures of proteins. The initial structure was then improved for geometry and accuracy of the atomic positions [33].

#### 4.7.3. Geometric Validation

The geometric validity and stereochemical accuracy of the improved structure were checked using SAVES v6.1 (https://saves.mbi.ucla.edu), mainly relying on the validation tool PROCHECK. This gave us a Ramachandran plot that helped verify that the dihedral angles are correctly distributed in the most favored and allowed regions [34].

### 4.8. Molecular Docking with Human TLR4 Receptor

To assess the level of interaction of the vaccine construct with the innate immune system, protein–protein docking was performed using the ClusPro 2.0 program (https://cluspro.org/help.php) (accessed on 14 March 2026). The previously validated 3D structure of the vaccine was docked with the human Toll-like Receptor 4 (TLR4) receptor. Docking results were ranked with the ClusPro weighted scoring function, which combines the van der Waals, electrostatic, and desolvation energy terms in favor of the most likely binding conformations. These weighted scores are dimensionless ranking scores and should not be viewed as absolute binding free energies or thermodynamic measures [35,36,37].

### 4.9. Protein–Ligand Interaction Profiling

The best docked complex was further investigated using PyMOL version 2.5.2 (https://www.pymol.org), where the precise nature of the interaction was determined. The interaction profiling was carried out based on hydrogen bonds, polar, and hydrophobic interactions within a cutoff of 4 Å. The profiling was further used to identify the receptor sites of the TLR4 receptor responsible for the stable interaction with the adjuvant and epitope regions of the vaccine [35,36].

### 4.10. Global Population Coverage Analysis

To understand the efficacy of the vaccine in various ethnic groups and geographical locations, a global population coverage analysis was carried out with the IEDB database (https://tools.iedb.org/population) (accessed on 15 March 2026). The identified MHC Class I and II epitopes of the vaccine candidate were compared with the various racial and ethnic groups of the global population based on the frequency of the HLA alleles. Analysis was conducted with the IEDB global population dataset and the coverage hence represents an aggregate estimate of the frequencies of the alleles reported in this dataset, and not uniform coverage of all geographic or ethnic populations [38].

### 4.11. Normal Mode Analysis (NMA) and Structural Stability

The motion of the vaccine–TLR4 complex was determined using the Normal Mode Analysis (NMA) method with the iMODS server (https://imods.iqf.csic.es). The Normal Mode Analysis provided information about the flexibility of the proteins by calculating the eigenvalues, B-factors, and the deformability of the proteins. By using the Normal Mode Analysis method, the stability of the docked complex was determined, ensuring the stability of the complex in the physiological state of the cell [39].

### 4.12. Immune Response Dynamics

The immunogenicity of the multi-epitope vaccine was simulated using the C-IMMSIM server (https://kraken.iac.rm.cnr.it/C-IMMSIM/index.php) (accessed on 17 March 2026). The immunogenicity of the vaccine was determined using Parker’s propensity scale for the prediction of B-cell epitopes. The immune kinetics of the vaccine were simulated for a period of 12 weeks, and the levels of IgG and IgM antibodies, T-cell population, and cytokine levels such as IFN-γ and IL-2 were determined, indicating the immunogenicity of the vaccine candidate [40,41,42].

### 4.13. Reverse Translation and Codon Optimization

The amino acid sequence of the designed multi-epitope vaccine construct was reverse translated to a nucleotide sequence by using the Reverse Translate tool of the Sequence Manipulation Suite (SMS) [43]. This resulted in a coding sequence which was then optimized for heterologous expression in *E. coli* K-12 with the VectorBuilder’s codon optimization tool. The codon-usage preference of the host was accounted for by codon optimization to maximize compatibility without altering the original amino acid sequence of the recombinant, or sequences which may be detrimental for recombinant expression [44].

## 5. Conclusions

In this study, a rationally designed multi-epitope vaccine candidate against Batai orthobunyavirus envelope glycoprotein was developed by immunoinformatics and structural modeling. The construct exhibited a positive predicted antigenicity, structural stability, and wide population coverage, as well as in silico simulations and immune response. The results presented here offer the computational basis for additional tests to be conducted regarding the proposed immunogenicity, receptor-mediated signaling, and protective activity; however, in vitro and in vivo tests must be carried out prior to any definitive conclusions regarding the immunogenicity, receptor-mediated signaling, and protection.

## Figures and Tables

**Table 1:** The physicochemical properties of the candidate glycoprotein for vaccine design.

Property	Value
Number of Amino Acids	1434
Molecular Weight (kDa)	162.19
Theoretical Isoelectric Point (pI)	7.68
Total Number of Atoms	22,738
Instability Index (II)	39.86 (Stable)
Aliphatic Index	88.02
Grand Average of Hydropathicity (GRAVY)	−0.099 (Hydrophilic)
Estimated Half-life (Mammalian reticulocytes, in vitro)	30 h
Extinction Coefficient	174,785
Negatively Charged Residues (Asp + Glu)	156
Positively Charged Residues (Arg + Lys)	160

**Table 2:** The selected B-cell epitopes utilized in the vaccine construction.

No.	Position	Sequence	Length	Flexibility Score	Hydrophilicity Percentage	Surface Area
1	16–38	SPVVTRCFHGGQLIAEKKSQTAV	23	1.125	68.40%	1.24
2	277–296	EASDRMRIHRESGLCQGYKS	20	1.085	72.10%	1.195
3	329–347	EGTLTNYPESRKYDLEEIA	19	0.985	65.00%	1.08
4	819–834	CFLTEPEVSDDVFSIA	16	1.24	78.50%	1.35
5	905–924	EYHSRKSKYITSLDLESLEE	20	1.01	64.20%	1.11

**Table 3:** The selected MHC Class I (CTL) epitopes based on the IC50 < 10 utilized in the vaccine construction.

No.	Sequence	GRAVY Score	MHC Allele	Length
1	MPLFMPIAY	1.356	HLA-A*02:01	9-mer
2	MPIAYFYGW	0.611	HLA-A*24:02	
3	ETIDVANVR	−0.111	HLA-A*03:01	
4	KSNNKGEVW	−1.8	HLA-B*07:02	
5	FTSYSCSFK	−0.022	HLA-A*11:01	
6	LLMALVIIV	3.611	HLA-A*02:01	
7	AQLAKILEV	1	HLA-A*02:01	
8	LLMPLFMPI	2.144	HLA-A*02:01	
9	FAGEMNSTY	−0.411	HLA-A*24:02	
10	FPLFDIIIF	2.289	HLA-A*02:01	
11	ALFLLMALV	3.078	HLA-A*02:01	
12	IYIFLPMVF	2.4	HLA-A*02:01	
13	KVLRNWIIK	0.033	HLA-A*03:01	
14	FQTEKLNHF	−0.989	HLA-B*44:03	
15	HTLIVYNFK	0.3	HLA-A*11:01	
16	LAIYIFLPM	2.244	HLA-A*02:01	
17	KSKECGHAK	−1.7	HLA-A*03:01	

**Table 4:** The selected MHC Class II (HTL) epitopes based on the IC50 < 10 utilized in the vaccine construction with hydrophilicity and allele specifications.

No.	Sequence	GRAVY Score	MHC Allele	Length
1	CQGYKSLRVARLLCK	−0.027	HLA-DRB1*01:01	15-mer
2	GALFLLMALVIIVAL	3.073	HLA-DRB1*04:01	
3	VVSIIVLFLAIYIFL	3.153	HLA-DRB1*15:01	
4	LQFKLMLGDLRYKSF	0.06	HLA-DRB1*07:01	
5	QNIELIIIISLTLII	2.06	HLA-DRB1*11:01	
6	YTSIFGALFLLMALV	2.12	HLA-DRB1*03:01	
7	IAFYVVVSIIVLFLA	3	HLA-DRB1*13:01	
8	LIISILLSMLILSFV	2.9	HLA-DRB1*04:04	
9	FPLFDIIIFIKSAII	2.08	HLA-DRB1*01:01	
10	SLEEFKRAISEKLSH	−0.78	HLA-DRB1*15:01	

**Table 5:** Detailed interaction profile of the human TLR4 receptor residues with the BATV vaccine construct.

Interaction Category	Specific TLR4 Receptor Residues
Basic (Positively Charged)	Lys47, Arg67, Arg87, Arg264
Acidic (Negatively Charged)	Glu42, Glu89, Glu94, Asp238, Glu266, Glu270, Asp294
Polar (Uncharged)	Asn44, Tyr46, His68, Ser71, Ser73, Gln91, Gln115, Asn137, Gln188, Asn216, Asn241, Asn265, Asn268
Hydrophobic/Non-polar	Pro28, Leu43, Ile48, Phe63, Pro65, Leu66, Gly70, Pro113, Gly267, Tyr292

**Table 6:** The simulation timepoints at 12 week interval.

Timepoint	Day	IgM (Ab Titer)	IgG1 + IgG2 (Ab Titer)	Memory B-cells	IFN-γ (ng/mL)	IL-2 (ng/mL)
Week 1	7	696.3	159.8	107.4	351,370	102,959
Week 4	28	439.5	977.9	112.9	24,658	1420
Week 8	56	60.9	405.3	110.5	2615	0
Week 12	84	7.6	137.0	102.3	0	0

## Data Availability

The original contributions presented in this study are included in the article/Supplementary material. Further inquiries can be directed to the corresponding author.

## Supplementary Materials

The following supporting information can be downloaded at: https://www.mdpi.com/article/10.3390/ph19081307/s1. Table S1: Raw data of B-Cell Epitopes Prediction; Table S2: Raw data of predicted CTL Epitopes and their percentile ranks; Table S3: Raw data of predicted HTL Epitopes and their percentile ranks; Table S4: The vaccine construction architecture of the designed construct.
